# Mössbauer Study on the Conversion of Different Iron-Based Catalysts Used in Carbon Nanotube Synthesis

**DOI:** 10.3390/nano13233010

**Published:** 2023-11-23

**Authors:** Michal Kořenek, Tatiana Ivanova, Veronika Svačinová, Miroslav Mašláň

**Affiliations:** Faculty of Science, Palacký University, 17. Listopadu 1192/12, 77900 Olomouc, Czech Republicmiroslav.maslan@upol.cz (M.M.)

**Keywords:** iron catalyst, iron transformation, carbon encapsulation, carbon nanotubes, chemical vapour deposition, Mössbauer spectroscopy, X-ray powder diffraction

## Abstract

The phase composition and comparison of iron-based catalysts used for the synthesis of carbon nanotubes were investigated. This work reflects typical catalyst conditions and their evolution during the growth of carbon nanotubes. The preparation of carbon nanotubes was carried out by chemical vapour deposition at temperatures between 800 and 1100 °C. Ferrocene or zero-valent iron nanoparticles were used as “catalysts”, and toluene, ferrocene and the ferrocene–toluene solution played the role of carbon precursors, respectively. The phase composition of the prepared product was studied by Mössbauer spectroscopy and X-ray powder diffraction. Mössbauer analysis was particularly useful for samples with a low content of the nanoparticle form of the catalyst. The composition of the prepared samples differed depending on the synthesis temperature, catalyst and precursor. Phase analysis revealed the presence of α-Fe and Fe_3_C in all samples. In addition, γ-Fe and iron oxides were identified under certain conditions. Scanning and transmission electron microscopy confirmed the carbon nanotube/nanofibre-like morphology and the presence of iron species.

## 1. Introduction

Carbon nanotubes (CNTs) have attracted much attention due to their interesting combination of properties such as tensile strength, electrical and thermal conductivity, chemical resistance and relatively high surface area [1]. The properties of the prepared CNTs can significantly vary because of different synthetic approaches, methodologies or simply reaction conditions. The differences in CNTs’ characteristics are mainly observed in size distribution, their internal morphology, electrical properties, etc. Over the years, the application of CNTs has been imagined in many fields: from the use of single nanotubes for biological and medical purposes [2,3], across nanoscale electronics [4,5] and energy storage [6,7], to ‘bulk’ additives in civil engineering and industry [8,9]. Needless to say, the mass production of CNTs is still limited as even a small alteration in the synthetic protocol can have severe effects on the product’s properties [10]. In chemical vapour deposition reaction, i.e., the most commonly used technique for CNT preparation, the most important outcome-determining factors are temperature [11,12,13,14], carbon precursor (source) [13,14,15,16] and the choice of a so-called “catalyst”.

The catalyst role is most often played by metal nanoparticles, which provide a significant increase in the CNTs’ yield and effectively stimulate CNTs’ growth. The catalytic activity of the catalyst material depends on many factors, including carbon solubility, diffusion rate inside these materials, size and shape of the catalytic material, etc. [17]. For example, in the case of Pt, Pd, Mn, Mo, Cr, Sn, Au, Mg and Al, which have all been used as catalysts, the size of the “catalyst” particle appears to be critical [18]. Noble metals such as Au, Pt and Pd have one of the lowest carbon solubilities, but their nanoparticle form (<5 nm in diameter) can dissolve carbon in sufficient quantities to support CNT growth as reported in [19]. Nonetheless, the most commonly used catalysts for carbon nanotube synthesis are Fe-, Co- or Ni-based materials [20]. Among these, Fe catalysts stand out for their high solubility of carbon, the possibility to use different morphologies, their great abundance on Earth, their environmental friendliness and their relatively low price. In the last few decades, much effort has been made to map the influence of various catalysts on the CNTs’ growth. However, so far, only a few authors have been interested in the fate of these catalysts after the synthesis. The catalyst material often stays embedded in the carbon material and needs to be removed if the pure CNTs are the final product. However, despite being called catalysts, these metal nanoparticles most often undergo phase transformations during the CNTs’ growth [21]. This, if properly controlled, could give rise to new nanocomposite materials, where metallic, oxidic or carbide nanoparticles are embedded inside the carbon matrix. Such materials hold great promise in numerous applications, but especially in heterogenous catalysis [22,23].

In this paper, we present a Mössbauer investigation of the various iron-based “catalyst” particles after CNTs’ synthesis via CVD deposition. Mössbauer spectroscopy is a technique of high resolution (neV) and can provide valuable insight into the phase transformation of the “catalyst” particles during the reactions. Thanks to such resolution, it may be possible to observe tiny particles that are within the limits of other techniques, e.g., measurement of small nanoparticles with X-ray powder diffraction [24]. Several Mössbauer studies have already been presented in the past. However, the studies were focused on different aspects, such as a study of the spatial distribution of the catalyst along the aligned CNTs’ forests [25] or others [26,27]. We seek to provide a direct comparison between the different situations and their outcomes that are commonly encountered in CNTs’ preparation, i.e., synthesis (i) with a sufficient source of carbon, (ii) with an insufficient amount of carbon and (iii) with a partially oxidised catalyst. The comparison of results among the available data is not always straightforward as the results are influenced by secondary factors, e.g., a different laboratory apparatus, conditions, modes (CVD, laser ablation, arc discharge), etc. 

Here, all the reactions were carried out at several temperatures between 800 and 1100 °C, which are especially interesting for the typical α/γ iron lattice transitions and allow for the full decomposition of the used carbon precursors (sources). Ferrocene (both solid or in solution with toluene) and passivated zero-valent iron nanoparticles were employed as the “catalysts” whose phase transitions were investigated. Mössbauer spectroscopy was further supplemented with XRD and scanning and transmission electron microscopy.

## 2. Materials and Methods

**Figure 1:**
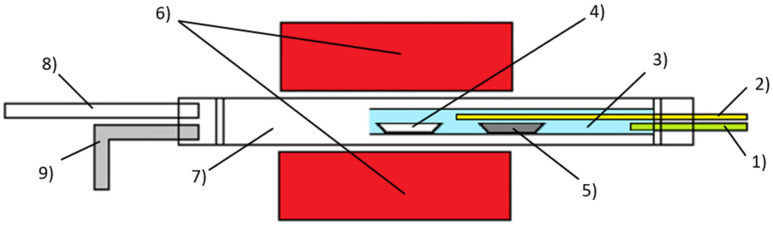
Chemical vapour deposition setup. (1) Inert gas inlet, (2) capillary for precursor injection, (3) quartz tube, (4) ceramic plate with ZVI nanoparticle catalyst, (5) ceramic plate with solid ferrocene, (6) cylindrical furnace with a hollow core, (7) stainless steel tube, (8) pump outlet and (9) gas outlet.

The carbon nanotubes were synthesised by chemical vapour deposition (CVD). The synthesis was carried out in a Nabertherm stainless steel tube furnace (Nabertherm GmbH, Lilienthal, Germany) with an inner quartz tube. The CVD laboratory equipment is shown in Figure 1. In the first case, a ferrocene–toluene (F + T) solution with a concentration of 35 mg/mL was prepared and 5 mL of the solution was used in the synthesis. This solution was injected into the workspace via a capillary using a Ne-1000 syringe dispenser. The synthesised CNTs were deposited in a ceramic dish placed at the end of a quartz tube in the centre of the furnace heating zone. In the second case, 500 mg of ferrocene (purity 99+%, Alfa Aesar, Haverhill, MA, USA) was placed in a ceramic dish outside the centre of the furnace heating zone, where the temperature was sufficient to evaporate the ferrocene. In the third case, a ceramic dish containing 100 mg of zero-valent iron (ZVI) nanoparticles (NANOFER STAR from NANOIRON s.r.o., Židlochovice, Czech Republic) was placed in the centre of the reaction chamber and 5 mL of toluene (from Penta s.r.o., Praha, Czech Republic) was injected through the capillary. The liquid carbonaceous precursor was injected at a rate of 10 mL/h after reaching the selected temperature. The synthesis was carried out at temperatures between 800 and 1100 °C. The temperature rise took 30 min and the temperature was maintained at the set temperature for a further 30 min. The reaction chamber was spontaneously cooled in approximately 6 h. The synthesis was carried out under an inert Ar atmosphere of 1.5 bar.

The phase composition of the prepared products was analysed using a custom-built transmission Mössbauer spectrometer described in [28] with a ^57^Co(Rh) source and a Bruker D8 ADVANCE X-ray powder diffractometer (Bruker, Billerica, MA, USA) equipped with a Co Kα X-ray source and an LYNXEYE position-sensitive detector. The diffractometer operates with Bragg–Brentano parafocusing geometry. The X-ray tube current and voltage were 40 mA and 35 kV, respectively. Measurements were made in the 2θ range of 15–120° with a step of 0.02° and a time per step of 3.00 s. The diffractometer was equipped with a 0.6 mm divergence slit and 2.5° axial Soller slits for the primary beam path and a 20 µm Fe Kβ filter and 2.5° axial Soller slits for the secondary beam path. The least squares fitting of the lines using the MossWinn 4.0 software program [29] was used to calculate and evaluate the Mössbauer spectra. The isomer shift values were referenced to the centroid of the spectrum recorded from an α-Fe foil (thickness 30 µm) at room temperature. The XRD patterns were evaluated using DIFFRAC.EVA v5.1.0.5 software with Crystallography Open Database (COD). The phases were evaluated by carbon—COD9008569, Fe_3_C—COD9012187, α-Fe—COD1100108, γ-Fe—COD9008469 and α-Fe_2_O_3_—COD2108027. The morphology of the products was analysed by scanning electron microscope VEGA3 (TESCAN, Brno, Czech Republic) with XFLASH silicon drift detector 410-M (Bruker Nano GmbH, Berlin, Germany) for energy dispersive spectroscopy and transmission electron microscopes (LVEM5D and JEOL-NEOARM).

## 3. Results and Discussion

### 3.1. Mössbauer Spectroscopy

#### 3.1.1. CNTs Synthesised from F + T Solution

**Figure 2:**
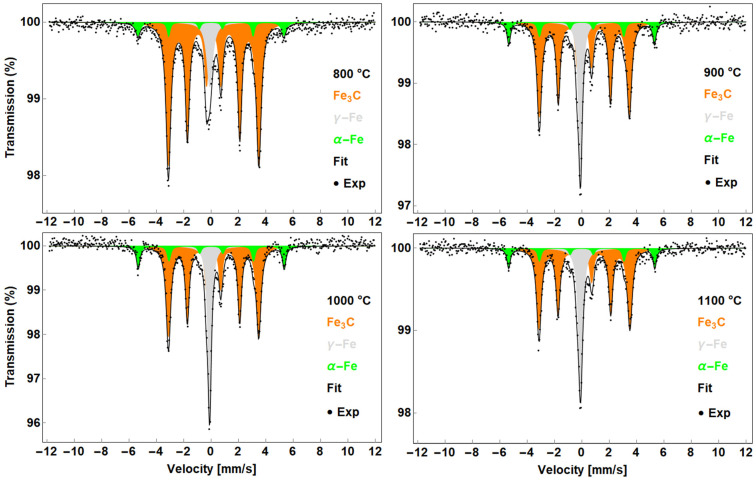
Mössbauer spectra of the samples prepared from F + T solution.

The Mössbauer spectra of the products prepared from the F + T solution are shown in Figure 2. The spectra were deconvoluted into a singlet and two sextets. Based on the hyperfine parameters summarised in Table 1, the singlet is referred to as the γ-Fe with a typical isomer shift of −0.1 mm/s. The sextets with magnetic hyperfine splitting 33.1 and 20.5 T are referred to as the α-Fe and Fe_3_C, respectively.

Higher iron carbides were also considered, but the hyperfine parameters indicate the presence of cementite. Theoretically, Fe_3_C should be described as two sextets with magnetic hyperfine splitting 20.5 and 20.7 T. However, such a close position is often approximated by a single sextet with a broader distribution [30]. Iron oxides were not identified in these samples, suggesting that all iron nanoparticles are completely covered by a carbon shell protecting them from oxidation. Given the hyperfine parameters and line widths, it is suggested that γ-Fe can be represented as an iron lattice with interstitial carbon atoms, similar to the iron lattice in steels [31]. Interestingly, the α-Fe parameters do not indicate the presence of carbon in the structure. A similar result was observed for 800 °C products in [32]. The presence of γ-Fe can be reflected by the Fe-C binary diagram as proposed previously [21]. Based on the temperature level used for synthesis and the transition temperatures in the diagram, γ-Fe or γ-Fe/Fe_x_C_y_ nanoparticles are most likely formed during synthesis. Further decomposition of the precursor hydrocarbons provides additional carbon atoms to support the growth of the carbon filaments. It is believed that during the cooling phase, the trapped particles in closed regions such as the tip or centre of the grown carbon nanotube remain in the face-centred cubic (fcc) lattice and do not transform into the more stable body-centred cubic (bcc) structure. Interestingly, the phase composition (A) estimated from the Mössbauer spectra shows almost the same ratio of each phase above 900 °C. Fe_3_C is shown as the main phase. Subsequently, α-Fe would represent the fraction of transformed particles and γ-Fe would represent the trapped particles. Based on the in situ measurement [33], the iron carbides are considered as intermediate phases when the hydrocarbon molecule is decomposed on the surface of the iron catalyst and remains as stable Fe_3_C after the end of precursor access. Although the amount of carbon nanotubes was observed to be significantly lower at 800 °C, no iron oxide was identified.

#### 3.1.2. CNTs Synthesised from Solid Ferrocene

**Figure 3:**
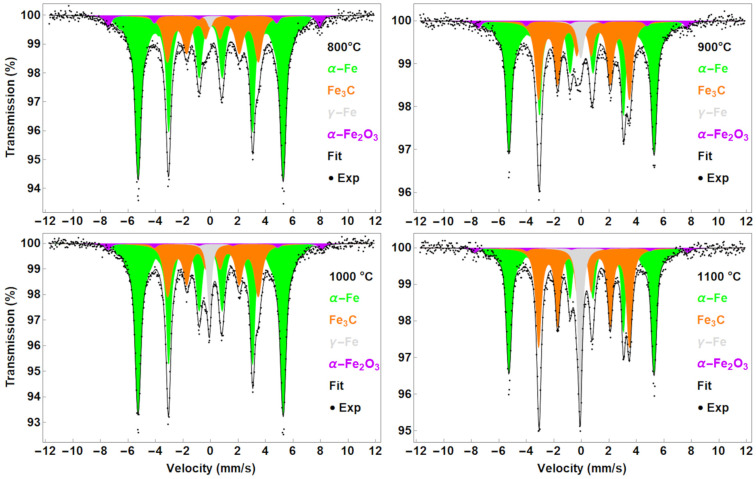
Mössbauer spectra of the samples prepared from the solid ferrocene.

**Figure 4:**
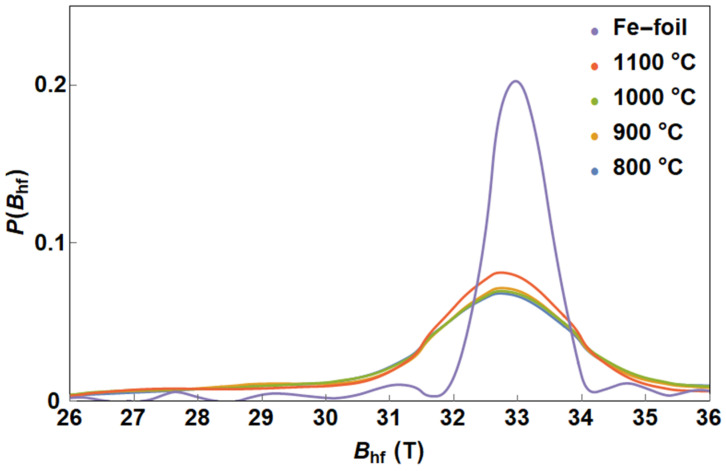
Distribution of hyperfine magnetic fields.

The products prepared from solid ferrocene show a case where a larger amount of iron is present compared to the carbon source. The Mössbauer spectra revealed different situations in the catalyst composition. The dominant sextet, termed α-Fe (Figure 3, Table 2), can be interpreted as a solid solution of carbon in the α-Fe crystal lattice, with carbon atoms occupying different interstitial positions in the crystal lattice. A consequence of the presence of carbon in the bcc α-Fe crystal lattice is the broadening of the distribution of hyperfine magnetic fields. Figure 4 compares the distribution of the hyperfine magnetic fields with the hyperfine field distribution in a pure α-Fe film. The distribution profile of the hyperfine magnetic fields is the same for the samples synthesised at 800 °C, 900 °C and 1000 °C. A slight increase in intensity at the maximum occurs at a synthesis temperature of 1100 °C. The position of the maximum of the distributions is in all cases identical to that in pure α-Fe.

**Figure 7:**
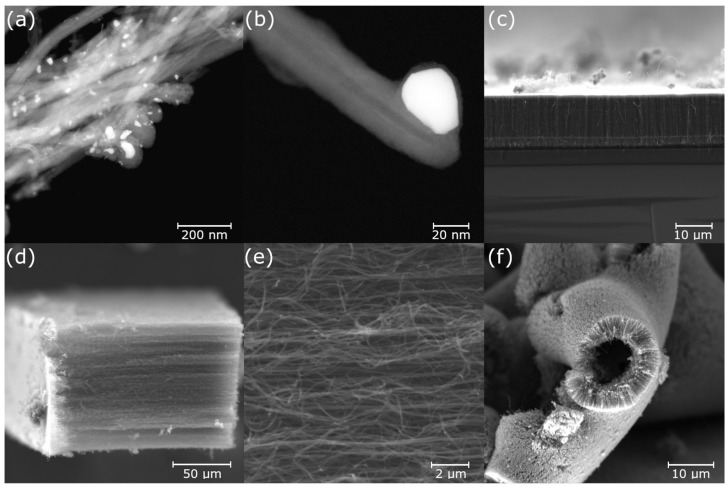
TEM images of carbon nanotubes prepared with F + T at 1000 °C (**a**,**b**), SEM images of CNTs prepared from F + T at 900 and 1000 °C (**d**,**e**) and CNTs prepared with solid ferrocene at 900 °C (**c**) and 1100 °C (**f**).

In this case, α-Fe represents the majority, but its amount, like that of Fe_3_C, varies non-trivially. This suggests that parameters other than temperature may play a role in the synthesis. The presence of Fe_3_C was also confirmed in all samples. On the other hand, the presence of γ-Fe is not clear up to 900 °C. In addition, SEM measurements showed different sizes of CNTs synthesised from the F + T solution and solid ferrocene (see Figure 7, Supplementary Figure S6). It is possible that these “spheres” are formed by trapped α-Fe/Fe_3_C particles and that γ-Fe particles exist only inside the nanotube structure (Supplementary Figures S6 and S7). The signal (Mössbauer spectra) is therefore mostly related to surface particles rather than particles inside the structure. Almost 90% of the abundance is presented by α-Fe and Fe_3_C. The amount of γ-Fe increases with temperature as shown in Figure 3. The hyperfine parameters of γ-Fe below 900 °C may be inaccurate due to the small amount. In addition, the α-Fe_2_O_3_ sextet [34] was detected. Even though the identification of α-Fe_2_O_3_ is near the limit of error, it seems to be presented in all samples, especially visible in the 800 °C sample. Moreover, the XRD pattern revealed a tiny reflection at 41° that suggests the presence of iron oxide. Its presence also reflects the partial oxidation of the starting solid ferrocene (see Supplementary Materials). 

#### 3.1.3. CNTs Synthesised with ZVI Nanoparticle Catalyst

**Figure 5:**
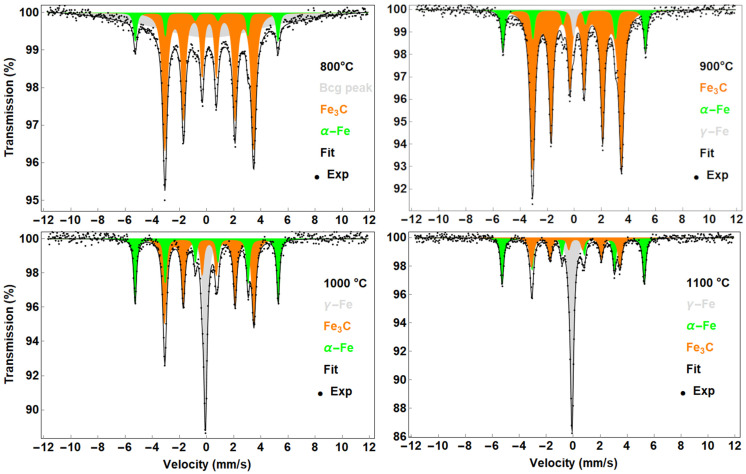
Mössbauer spectra of the samples prepared with ZVI nanoparticles as catalyst.

ZVI nanoparticles show a case where a mildly oxidised catalyst was used in the synthesis. The ZVI nanoparticles are pyrolytic but have been previously passivated by the manufacturer with a thin protective layer of iron oxide. Further details can be found in the Supplementary Materials. During synthesis in the presence of an iron oxide catalyst, the iron oxide is reduced and subsequently CNT growth occurs. However, suppose that the amount of carbon is insufficient. In that case, residual oxides may remain in the sample (see Supplement Figures S3 and S4) as it is determined by a “broad background” (Bcg peak in Figure 5—800 °C). Moreover, in detailed XRD analysis, the indication is not as clear as in previous solid ferrocene cases and overlap with Fe_3_C reflection occurs (Figure 5). Therefore, nanostructured or amorphous form of the residual phase can be presented. This was not observed at higher temperatures. In all samples, two sextets with magnetic hyperfine splitting of 32.7 and 20.3 T were identified as α-Fe and Fe_3_C, respectively (Table 3). The presence of carbon slightly reduced the magnetic hyperfine splitting of the α-Fe phase. When synthesised above 900 °C, γ-Fe was clearly identified, the proportion of which showed an increasing trend with temperature and a maximum at 1100 °C. This is related to the transformation of α-Fe into γ-Fe, which, according to the Fe-C phase diagram, occurs at temperatures above 900 °C and decreases slightly with increasing carbon content.

### 3.2. X-ray Powder Diffraction

**Figure 6:**
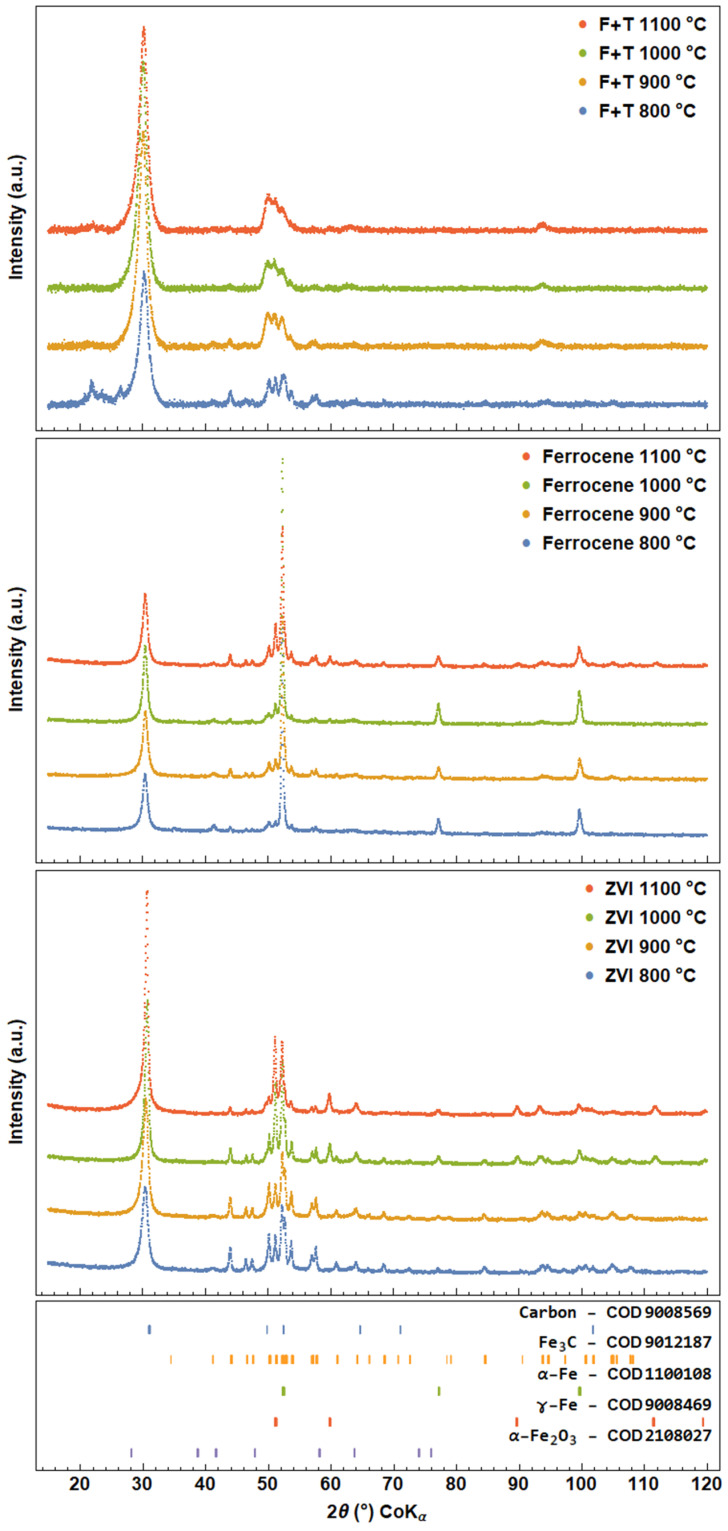
X-ray powder diffraction patterns. F + T represents the products from ferrocene–toluene solution, ferrocene represents the products from solid ferrocene and ZVI represents the products from zero-valent iron nanoparticles. The COD number refers to the phase structure in the Crystallography Open Database.

X-ray powder diffraction supported the information from Mössbauer spectroscopy. All XRD patterns contain two main regions as shown in Figure 6. The first region is a broad peak at 30.5° of the 2θ(Co)Kα region, which can be identified as the (002) diffraction peak of carbon. In the second region, the peaks in the 50–55° region appear to be composed of several phase reflections—carbon (101) and (100), γ-Fe (111), α-Fe (110) and carbide (211), (102), (220), (031) and (112). A wide range of XRD patterns can clarify the description of the phases shown. 

Upon detailed analysis, the peak profile at 30.5° is asymmetric, which could be caused by several factors, including diameter distribution, structural defects, curvature, nanotube orientation and the presence of graphite as described in [35]. It has been observed that the intraplane distance of carbon nanotubes decreases with increasing number of walls, starting from 3.8 Å for DWCNTs to 3.2 Å for MWCNTs with diameters > 30 nm. In our case, the wide diameter distribution, possible presence of graphite and structural defects most likely caused the broadening and asymmetry of the carbon peak. In addition, the volume of the product increases with increasing synthesis temperature, indicating possible changes in composition. This is particularly evident in the cases of CNT synthesis from the F + T solution and with the ZVI catalyst. 

The second region (52–55°) varies depending on the catalyst used and the temperature, confirming the results obtained by Mössbauer spectroscopy. In the F + T synthesis, the peaks in this region merge more with increasing temperature. The presence of Fe_3_C and carbon can be well determined at 800 °C, but the presence of γ-Fe and α-Fe is speculative. As the temperature increases, the side peaks of Fe_3_C at 44° and 57.5° decrease, corresponding to the decrease in Fe_3_C observed in the Mössbauer measurement. In contrast, MS shows a higher formation of γ-Fe and α-Fe phases above 800 °C. Although the abundances of γ-Fe and α-Fe remain much lower compared to Fe_3_C and carbon, their nanoparticle form could contribute to the blurring of the 50–55° region. Interestingly, a controversy arises with a peak at 94.5°. The increasing amount of carbon indicates the increase in carbon reflections against the intensity of the catalyst. However, if we consider the increase in intensity at 50° due to the carbon reflection (101), the peak at 94.5° is not consistent with the lattice parameter of carbon and its possible (110) reflection at this position without any texture effect. As a more favourable result, the 94.5° position is attributed to a series of reflections from Fe_3_C, although its abundance has decreased relative to 800 °C as determined by Mössbauer spectroscopy. The peak in the foreground at 800 °C remains unknown. A similar peak appeared in [36,37] but was not identified here either.

In the case of the solid catalysts (solid ferrocene, ZVI nanoparticles), the amount of the iron-based phase is greater, resulting in a higher peak resolution. For example, α-Fe is easily recognisable in the ferrocene products by its positions at 52.2°, 77° and 100°. In the case of the ferrocene catalyst, the presence of α-Fe_2_O_3_ at 800 °C was suggested by the (110) reflection at 41.4°. The tiny peak at 41.4° also remained above 800 °C, suggesting the possible presence of iron oxide, which is related to the α-Fe_2_O_3_ in Mössbauer spectra (Figure 3). However, the possible overlap with the (200) reflection of Fe_3_C should be considered.

In the case of the ZVI catalyst, Fe_3_C plays a great role in the form of iron. Its abundance decreases with increasing temperature. This is in contrast to the fitted MS spectrum at 800 °C, where the amount of Fe_3_C is initially low. The intense broad singlet in the Mössbauer spectrum is probably overestimated, while the α-Fe and iron carbide phases are depleted. The (110) α-Fe reflection is noticeable because the peak at 52.3° is higher than others in this region. Based on the XRD results, the singlet should be split into α-Fe and Fe_3_C. As the synthesis temperature increases, the amount of γ-Fe increases. In contrast to MS, the amount of γ-Fe in the XRD fit does not exceed that of Fe_3_C, indicating small crystal domains and small nanoparticles of γ-Fe.

### 3.3. Electron Microscopy

Based on the information obtained from the scanning electron microscope (SEM), the presence of carbon filament nanostructures was confirmed. Detailed transmission electron microscopy analysis revealed the structure of multi-walled CNTs. The estimated diameter of the carbon nanotubes ranged from 20 to 100 nm (Figure 7a,b) depending on the experimental conditions. The wide-diameter distribution is probably related to the free catalyst and its possible agglomeration or cleavage during synthesis.

The carbon nanotubes produced from the F + T solution were produced in sufficient quantities at temperatures above 800 °C, confirming the fact that the energy supplied is sufficient to decompose the carbon precursor (toluene) in the presence of the catalyst (ferrocene). The amount of CNTs increased with temperature. The presence of carbon in the form of carbon black chains and graphite films was also observed (Figure 7d,e). Interestingly, the carbon nanotubes grew in contact with the quartz tube in the form of CNT forests, and the carbon black chains were mainly located inside the ceramic tray into which the solution was injected. Transmission electron microscopy revealed the presence of catalytic iron inside the CNTs as shown in Figure 7a. The size of the catalytic particle appears to correspond to the diameter of the nanotube (Figure 7b) as reported in previous work [38,39]. Occasionally, part of the catalytic particle split during growth and became trapped in the hollow centre of the nanotube. Wall defects were found in the vicinity of the split particles. The mode of nanotube formation is likely to be the “tip growth” model described in [40]. 

In general, the amount of carbon in the solid ferrocene precursor is lower than in the F + T solution, resulting in a lower yield of CNTs. The greater amount of iron leads to the formation of iron–carbon phases rather than the oversaturation and growth of CNTs. Although the decomposition of ferrocene starts at 500 °C, the highest yield of CNTs was observed at 1100 °C. Surprisingly, the longest CNTs were observed at 1000 °C. The CNT forests grown at 1100 °C were shorter and rolled up as shown in Figure 7f.

**Figure 8:**
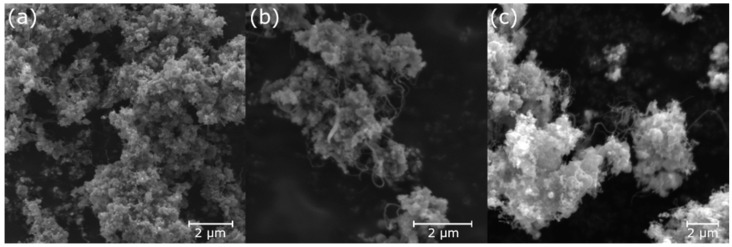
SEM images of carbon filaments prepared from toluene with ZVI nanoparticles as catalyst at (**a**) 800 °C, (**b**) 900 °C and (**c**) 1100 °C.

The ZVI nanoparticle catalyst is issued by its surface oxidic passivation layer. It is necessary to reduce iron oxides to make the catalyst active for CNT growth [41]. There are several approaches for iron oxide reduction. Hydrogen treatment can be applied before the carbon precursor is injected so that the catalyst is treated before the synthesis starts [42]. We were interested in the phase changes of ZVI nanoparticles and their passivation layer by the carbon precursor, so no hydrogen pre-treatment was applied. It is known that the hydrocarbon precursor itself can reduce such surface oxides during its decomposition. CNT growth begins after the particle is freed from the passivation layer and saturated with carbon atoms. However, if the iron oxide is not sufficiently reduced in the first moments of contact with a carbon precursor, a carbon shell can be formed and nanotube growth is stopped. Only a small amount of CNTs was observed within the resolution of the microscope. However, their presence was confirmed above 900 °C, with the highest density at 1100 °C. The CNTs are tens of microns long and have a diameter of up to 120 nm, suggesting aggregation of the nanoparticles during heating or agglomeration of the fibres during the growth process. The products are shown in Figure 8.

## 4. Conclusions

From the point of view of the transformation of iron catalysts for CNT growth, products prepared using the CVD method in the temperature range of 800 and 1100 °C were studied. CNTs were synthesised using three methods: (a) by the decomposition of toluene in which ferrocene was dissolved as a catalyst, (b) by the decomposition of toluene using zero-valent iron nanoparticles as a catalyst, and (c) by the decomposition of solid ferrocene. Each of these synthesis methods resulted in different amounts of α-Fe, γ-Fe and Fe_3_C in the final products and, of course, the synthesised CNTs also differed in character and size. 

In the study of such catalyst conversion, in which the majority of the sample is the carbon phase, the combination of such characterisation methods has proved necessary to determine the phase composition of the used catalyst. Mössbauer spectroscopy was used to identify different iron-containing phases in the final products. Depending on the type of CNT synthesis and the synthesis temperature, iron-containing phases were formed and transformed in different proportions. In all cases, the transformation from α-Fe to γ-Fe seemed to occur at temperatures above 900 °C. During the catalysis using zero-valent iron nanoparticles, the iron oxide passivating the nanoparticles was reduced by carbon. The iron oxide residue was observed in the Mössbauer spectrum of the product synthesised at 800 °C. In the case of the decomposition of solid ferrocenes, the most stable form of iron oxide (α-Fe_2_O_3_) was identified in trace amounts in all cases. The Mössbauer spectroscopy results were confirmed by XRD measurements.

## Figures and Tables

**Table 1:** Hyperfine parameters of the samples prepared from F + T solution (IS—isomer shift, FWHM—full width at half maximum, B—hyperfine magnetic field, A—spectrum area).

Temperature	Phase	IS (mm/s)	FWHM (mm/s)	B (T)	A (%)
800 °C	Fe_3_C	0.19 ± 0.01	^(3, 4)^ 0.34 ± 0.01	20.5 ± 0.5	82 ± 2
			^(2, 5)^ 0.35 ± 0.01		
			^(1, 6)^ 0.43 ± 0.01		
	α-Fe	0.01 ± 0.01	^(3, 4)^ 0.26 ± 0.07	33.1 ± 0.5	7 ± 2
			^(2, 5)^ 0.26 ± 0.04		
			^(1, 6)^ 0.41 ± 0.06		
	γ-Fe	−0.10 ± 0.01	0.51 ± 0.02	-	11 ± 2
900 °C	Fe_3_C	0.18 ± 0.01	^(3, 4)^ 0.29 ± 0.01	20.5 ± 0.5	67 ± 2
			^(2, 5)^ 0.32 ± 0.01		
			^(1, 6)^ 0.41 ± 0.01		
	α-Fe	0.02 ± 0.01	0.30 ± 0.03	33.1 ± 0.5	12 ± 2
	γ-Fe	−0.10 ± 0.01	0.32 ± 0.01	-	21 ± 2
1000 °C	Fe_3_C	0.18 ± 0.01	^(3, 4)^ 0.29 ± 0.01	20.5 ± 0.5	65 ± 2
			^(2, 5)^ 0.32 ± 0.01		
			^(1, 6)^ 0.38 ± 0.01		
	α-Fe	0.01 ± 0.01	0.28 *	33.3 ± 0.5	12 ± 2
	γ-Fe	−0.10 ± 0.01	0.38 ± 0.02	-	23 ± 2
1100 °C	Fe_3_C	0.19 ± 0.01	^(3, 4)^ 0.34 ± 0.02	20.7 ± 0.5	64 ± 2
			^(2, 5)^ 0.35 ± 0.01		
			^(1, 6)^ 0.42 ± 0.01		
	α-Fe	0.01 ± 0.01	0.29 ± 0.03	33.2 ± 0.5	11 ± 2
	γ-Fe	−0.09 ± 0.01	0.37 ± 0.01	-	25 ± 2

* indicates fixed values. ^(3, 4)^, ^(2, 5)^ and ^(1, 6)^ denote pairs of equally intense lines in certain sextet subspectra, the line position is determined from the left starting with 1.

**Table 2:** Hyperfine parameters of the samples prepared from the solid ferrocene. (IS—isomer shift, QS/ε—quadrupole splitting/shift, FWHM—full width at half maximum, B—hyperfine magnetic field, A—spectrum area).

Temperature	Phase	IS (mm/s)	QS/ε (mm/s)	FWHM (mm/s)	B (T)	A (%)
800 °C	Fe_3_C	0.16 ± 0.01	-	^(3, 4)^ 0.50 ± 0.04	20.5 ± 0.5	25 ± 2
				^(2, 5)^ 0.68 ± 0.03		
				^(1, 6)^ 0.65 ± 0.03		
	α-Fe	0.00 ± 0.01	-	^(3, 4)^ 0.47 ± 0.01	33.1 *	70 ± 2
				^(2, 5)^ 0.18 ± 0.03		
				^(1, 6)^ 0.17 ± 0.05		
	γ-Fe	−0.05 ± 0.01	-	0.44 ± 0.11	-	1 ± 2
	α-Fe_2_O_3_	0.30 ± 0.01	−0.10 ± 0.01	0.47 *	47.7 ± 0.5	4 ± 2
900 °C	Fe_3_C	0.18 ± 0.01	-	^(3, 4)^ 0.45 ± 0.03	20.5 ± 0.5	36 ± 2
				^(2, 5)^ 0.51 ± 0.01		
				^(1, 6)^ 0.54 ± 0.02		
	α-Fe	0.00 ± 0.01	-	^(3, 4)^ 0.44 ± 0.02	33.0 *	57 ± 2
				^(2, 5)^ 0.17 ± 0.04		
				^(1, 6)^ 0.17 ± 0.07		
	γ-Fe	−0.06 ± 0.01	-	0.51 ± 0.04	-	4 ± 2
	α-Fe_2_O_3_	0.23 ± 0.01	−0.29 ± 0.01	0.47 *	47.2 ± 0.5	3 ± 2
1000 °C	Fe_3_C	0.17 ± 0.01	-	^(3, 4)^ 0.50 ± 0.03	20.5 ± 0.5	25 ± 2
				^(2, 5)^ 0.60 ± 0.03		
				^(1, 6)^ 0.63 ± 0.02		
	α-Fe	0.00 ± 0.01	-	^(3, 4)^ 0.45 ± 0.01	33.0 *	68 ± 2
				^(2, 5)^ 0.17 ± 0.03		
				^(1, 6)^ 0.17 ± 0.05		
	γ-Fe	−0.09 ± 0.01	-	0.35 ± 0.01	-	5 ± 2
	α-Fe_2_O_3_	0.35 ± 0.01	−0.10 ± 0.01	0.47 *	48.1 ± 0.5	2 ± 2
1100 °C	Fe_3_C	0.18 ± 0.01	-	^(3, 4)^ 0.37 ± 0.02	20.5 ± 0.5	39 ± 2
				^(2, 5)^ 0.43 ± 0.01		
				^(1, 6)^ 0.46 ± 0.01		
	α-Fe	0.00 ± 0.01	-	^(3, 4)^ 0.37 ± 0.02	32.9 *	48 ± 2
				^(2, 5)^ 0.15 ± 0.04		
				^(1, 6)^ 0.16 ± 0.07		
	γ-Fe	−0.10 ± 0.01	-	0.34 ± 0.04	-	11 ± 2
	α-Fe_2_O_3_	0.24 ± 0.01	−0.52 ± 0.01	0.47 *	48.1 ± 0.5	2 ± 2

* indicates fixed values. ^(3, 4)^, ^(2, 5)^ and ^(1, 6)^ denote pairs of equally intense lines in certain sextet subspectra, the line position is determined from the left starting with 1.

**Table 3:** Hyperfine parameters of the samples prepared with ZVI nanoparticles as catalyst (IS—isomer shift, FWHM—full width at half maximum, B—hyperfine magnetic field, A—spectrum area).

Temperature	Phase	IS (mm/s)	FWHM (mm/s)	B (T)	A (%)
800 °C	α-Fe	−0.01 ± 0.01	^(3, 4)^ 0.33 ± 0.02	32.7 ± 0.5	5 ± 2
			^(2, 5)^ 0.22 ± 0.02		
			^(1, 6)^ 0.27 ± 0.02		
	Fe_3_C	0.19 ± 0.01	^(3, 4)^ 0.27 ± 0.01	20.3 ± 0.5	34 ± 2
			^(2, 5)^ 0.34 ± 0.01		
			^(1, 6)^ 0.39 ± 0.01		
	Bcg peak	0.20 ± 0.01	11.99 ± 0.54	-	61 ± 2
900 °C	α-Fe	−0.01 ± 0.01	^(3, 4)^ 0.27 ± 0.03	32.7 ± 0.5	14 ± 2
			^(2, 5)^ 0.27 ± 0.02		
			^(1, 6)^ 0.29 ± 0.01		
	Fe_3_C	0.19 ± 0.01	^(3, 4)^ 0.31 ± 0.01	20.4 ± 0.5	84 ± 2
			^(2, 5)^ 0.37 ± 0.01		
			^(1, 6)^ 0.44 ± 0.01		
	γ-Fe	−0.05 ± 0.01	0.30 *	-	2 ± 2
1000 °C	α-Fe	−0.00 ± 0.01	^(3, 4)^ 0.23 ± 0.02	32.8 ± 0.5	27 ± 2
			^(2, 5)^ 0.24 ± 0.01		
			^(1, 6)^ 0.25 ± 0.01		
	Fe_3_C	0.18 ± 0.01	^(3, 4)^ 0.28 ± 0.01	20.5 ± 0.5	50 ± 2
			^(2, 5)^ 0.30 ± 0.01		
			^(1, 6)^ 0.35 ± 0.01		
	γ-Fe	−0.09 ± 0.01	0.30 ± 0.01	-	23 ± 2
1100 °C	α-Fe	0.00 ± 0.01	^(3, 4)^ 0.25 ± 0.02	32.7 ± 0.5	35 ± 2
			^(2, 5)^ 0.27 ± 0.01		
			^(1, 6)^ 0.28 ± 0.01		
	Fe_3_C	0.19 ± 0.01	^(3, 4)^ 0.28 ± 0.03	20.4 ± 0.5	26 ± 2
			^(2, 5)^ 0.30 ± 0.01		
			^(1, 6)^ 0.33 ± 0.02		
	γ-Fe	−0.09 ± 0.01	0.30 ± 0.01	-	39 ± 2

* indicates fixed values. ^(3, 4)^, ^(2, 5)^ and ^(1, 6)^ denote pairs of equally intense lines in certain sextet subspectra, the line position is determined from the left starting with 1.

## Data Availability

The data presented in this study are available on request from the corresponding author.

## Supplementary Materials

The following supporting information can be downloaded at: https://www.mdpi.com/article/10.3390/nano13233010/s1, Figure S1. Mössbauer spectrum of the ferrocene; Figure S2. X-ray diffraction pattern of the ferrocene; Figure S3. Mössbauer spectrum of the as-prepared zerovalent iron nanoparticles; Figure S4. X-ray diffraction pattern of the as-prepared zerovalent iron nanoparticles; Figure S5. Image of the zerovalent iron nanoparticles from transmission electron microscope; Figure S6. SEM measurement of the products prepared from solid ferrocene; Figure S7. Low resolution TEM measurement of carbon nanotubes prepared by solid ferrocene; Table S1. Hyperfine parameters of the ferrocene; Table S2. Hyperfine parameters of the as-prepared zerovalent iron nanoparticles.

## References

[1] Harris P.J.F. (2009). Carbon Nanotube Science: Synthesis, Properties and Applications.

[2] Pei B., Wang W., Dunne N., Li X. (2019). Applications of Carbon Nanotubes in Bone Tissue Regeneration and Engineering: Superiority, Concerns, Current Advancements, and Prospects. Nanomaterials.

[3] Simon J., Flahaut E., Golzio M. (2019). Overview of Carbon Nanotubes for Biomedical Applications. Materials.

[4] Norizan M.N., Moklis M.H., Zulaikha S., Demon N., Halim N.A., Samsuri A., Mohamad I.S., Knight V.F., Abdullah N. (2020). Carbon Nanotubes: Functionalisation and Their Application in Chemical Sensors. RSC Adv..

[5] Peng L.-M., Zhang Z., Wang S. (2014). Carbon Nanotube Electronics: Recent Advances. Mater. Today.

[6] Sehrawat P., Julien C., Islam S.S. (2016). Carbon Nanotubes in Li-Ion Batteries: A Review. Mater. Sci. Eng. B.

[7] Ullah Rather S. (2020). Preparation, Characterization and Hydrogen Storage Studies of Carbon Nanotubes and Their Composites: A Review. Int. J. Hydrog. Energy.

[8] Nieto A., Budan J., Gupta R.K., Ansell T.Y. (2022). 3D Printed Carbon Nanotube Reinforced Stainless Steel via Selective Laser Melting. MRS Commun..

[9] Lu D., Shi X., Zhong J. (2022). Understanding the Role of Unzipped Carbon Nanotubes in Cement Pastes. Cem. Concr. Compos..

[10] Kim Y.A., Hayashi T., Endo M., Dresselhaus M.S., Vajtai R. (2013). Carbon Nanofibers. Springer Handbook of Nanomaterials.

[11] Ahmad M., Silva S.R.P. (2019). Low Temperature Growth of Carbon Nanotubes—A Review. Carbon.

[12] Hou G., Chauhan D., Ng V., Xu C., Yin Z., Paine M., Su R., Shanov V., Mast D., Schulz M. (2017). Gas Phase Pyrolysis Synthesis of Carbon Nanotubes at High Temperature. Mater. Des..

[13] Hata K., Futaba D.N., Mizuno K., Namai T., Yumura M., Iijima S. (2004). Water-Assisted Highly Efficient Synthesis of Impurity-Free Single-Walled Carbon Nanotubes. Science.

[14] Mayne M., Grobert N., Terrones M., Kamalakaran R., Rühle M., Kroto H.W., Walton D.R.M. (2001). Pyrolytic Production of Aligned Carbon Nanotubes from Homogeneously Dispersed Benzene-Based Aerosols. Chem. Phys. Lett..

[15] Paul S., Samdarshi S.K. (2011). A Green Precursor for Carbon Nanotube Synthesis. New Carbon Mater..

[16] Hiramatsu M., Hori M., Yellampalli S. (2011). Aligned Growth of Single-Walled and Double-Walled Carbon Nanotube Films by Control of Catalyst Preparation. Carbon Nanotubes—Synthesis, Characterization, Applications.

[17] Moisala A., Nasibulin A.G., Kauppinen E.I. (2003). The Role of Metal Nanoparticles in the Catalytic Production of Single-Walled Carbon—A Review. J. Phys. Condens. Matter.

[18] Kumar M., Yellampalli S. (2011). Carbon Nanotube Synthesis and Growth Mechanism. Carbon Nanotubes—Synthesis, Characterization, Applications.

[19] Yuan D., Ding L., Chu H., Feng Y., McNicholas T.P., Liu J. (2008). Horizontally Aligned Single-Walled Carbon Nanotube on Quartz from a Large Variety of Metal Catalysts. Nano Lett..

[20] de Jong K.P., Geus J.W. (2000). Carbon Nanofibers: Catalytic Synthesis and Applications. Catal. Rev..

[21] Deck C.P., Vecchio K. (2006). Prediction of Carbon Nanotube Growth Success by the Analysis of Carbon–Catalyst Binary Phase Diagrams. Carbon.

[22] Tian T., Cheng Y., Sun Z., Huang K., Lei M., Tang H. (2022). Carbon Nanotubes Supported Oxygen Reduction Reaction Catalysts: Role of Inner Tubes. Adv. Compos. Hybrid Mater..

[23] Gupta V.K., Saleh T.A. (2013). Sorption of Pollutants by Porous Carbon, Carbon Nanotubes and Fullerene—An Overview. Environ. Sci. Pollut. Res..

[24] Kumar R., Sahoo B. (2018). One-Step Pyrolytic Synthesis and Growth Mechanism of Core-Shell Type Fe/Fe 3 C-Graphite Nanoparticles-Embedded Carbon Globules. Nano-Struct. Nano-Objects.

[25] Ruskov T., Spirov I., Ritschel M., Müller C., Leonhardt A., Ruskov R. (2006). Mössbauer Morphological Analysis of Fe-Filled Multiwalled Carbon Nanotube Samples. J. Appl. Phys..

[26] Oshima H., Shimazu T., Siry M., Mibu K. (2009). Analysis of Fe Catalyst during Carbon Nanotube Synthesis by Mössbauer Spectroscopy. J. Phys. Chem. C.

[27] Mendonça F.G., Ardisson J.D., Rosmaninho M.G., Lago R.M., Tristão J.C. (2011). Mössbauer Study of Carbon Coated Iron Magnetic Nanoparticles Produced by Simultaneous Reduction/Pyrolysis. Hyperfine Interact..

[28] Stejskal A., Procházka V., Novák P., Dudka M. (2020). Mössbauer Spectrometer Designed for Measurements of Fast Processes. Nucl. Instrum. Methods Phys. Res. A.

[29] Klencsar Z., Kuzmann E., Vertes A. (1996). User-Friendly Software for Mössbauer Spectrum Analysis. J. Radioanal. Nucl. Chem..

[30] Sajitha E.P., Prasad V., Subramanyam S.V., Kumar Mishra A., Sarkar S., Bansal C. (2007). Structural, Magnetic and Mössbauer Studies of Iron Inclusions in a Carbon Matrix. J. Magn. Magn. Mater..

[31] Cook D.C. (1987). Strain Induced Martensite Formation in Stainless Steel. Metall. Trans. A.

[32] Lyubutin I.S., Anosova O.A., Frolov K.V., Sulyanov S.N., Okotrub A.V., Kudashov A.G., Bulusheva L.G. (2012). Iron Nanoparticles in Aligned Arrays of Pure and Nitrogen-Doped Carbon Nanotubes. Carbon.

[33] Baker R.T.K. (1989). Catalytic Growth of Carbon Filaments. Carbon.

[34] Zboril R., Mashlan M., Petridis D. (2002). Iron(III) oxides from thermal processes-synthesis, structural and magnetic properties, Mössbauer spectroscopy characterization, and applications. Chem. Mater..

[35] Futaba D.N., Yamada T., Kobashi K., Yumura M., Hata K. (2011). Macroscopic Wall Number Analysis of Single-Walled, Double-Walled, and Few-Walled Carbon Nanotubes by X-ray Diffraction. J. Am. Chem. Soc..

[36] Abdel-Ghani N.T., El-Chaghaby G.A., Helal F.S. (2015). Individual and Competitive Adsorption of Phenol and Nickel onto Multiwalled Carbon Nanotubes. J. Adv. Res..

[37] Ban F.Y., Majid S.R., Huang N.M., Lim H.N. (2012). Graphene Oxide and Its Electrochemical Performance. Int. J. Electrochem. Sci..

[38] Venkatesan S., Visvalingam B., Mannathusamy G., Viswanathan V., Rao A.G. (2018). Effect of Chemical Vapor Deposition Parameters on the Diameter of Multi-Walled Carbon Nanotubes. Int. Nano Lett..

[39] Pérez-Cabero M., Taboada J.B., Guerrero-Ruiz A., Overweg A.R., Rodríguez-Ramos I. (2006). The role of alpha-iron and cementite phases in the growing mechanism of carbon nanotubes: A 57Fe Mössbauer spectroscopy study. Phys. Chem. Chem. Phys..

[40] Venkataraman A., Amadi V., Chen Y., Papadopoulos C. (2019). Carbon Nanotube Assembly and Integration for Applications. Nanoscale Res. Lett..

[41] Lima M.D., Bonadiman R., De Andrade M.J., Toniolo J., Bergmann C.P. (2006). Synthesis of Multi-Walled Carbon Nanotubes by Catalytic Chemical Vapor Deposition Using Cr_2−x_Fe_x_O_3_ as Catalyst. Diam. Relat. Mater..

[42] Nessim G.D., Hart A.J., Kim J.S., Acquaviva D., Oh J., Morgan C.D., Seita M., Leib J.S., Thompson C.V. (2008). Tuning of Vertically-Aligned Carbon Nanotube Diameter and Areal Density through Catalyst Pre-Treatment. Nano Lett..

