# Supplementary material for: Mössbauer Study on the Conversion of Different Iron-Based Catalysts Used in Carbon Nanotube Synthesis
Source: Nanomaterials (Basel). 2023 Nov 23;13(23):3010. doi: 10.3390/nano13233010 (PMC10708456; doi:10.3390/nano13233010)
Supplement: Supplementary file 1 [file nanomaterials-13-03010-s001.zip › nanomaterials-2738361-supplementary.pdf]

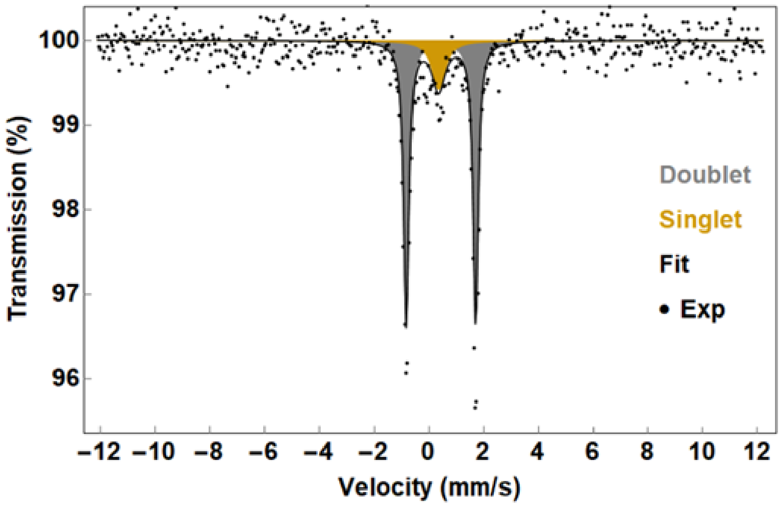

Figure S1. Mössbauer spectrum of the ferrocene.

Table S1. Hyperfine parameters of the ferrocene. (IS - isomer shift, QS – quadrupole splitting, FWHM - full width at half maximum, A – spectrum area).

Sample	Phase	IS (mm/s)	QS (mm/s)	FWMH (mm/s)	A (%)
**Ferrocene**	Doublet	0.44 ± 0.01	2.53 ± 0.01	0.23 ± 0.01	82 ± 2
	Singlet	0.36 ± 0.03	-	0.58 ± 0.10	18 ± 2

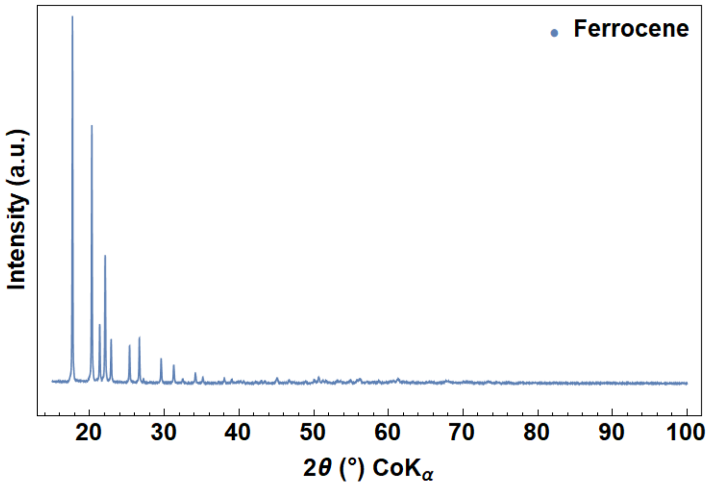

Figure S2. X-ray diffraction pattern of the ferrocene.

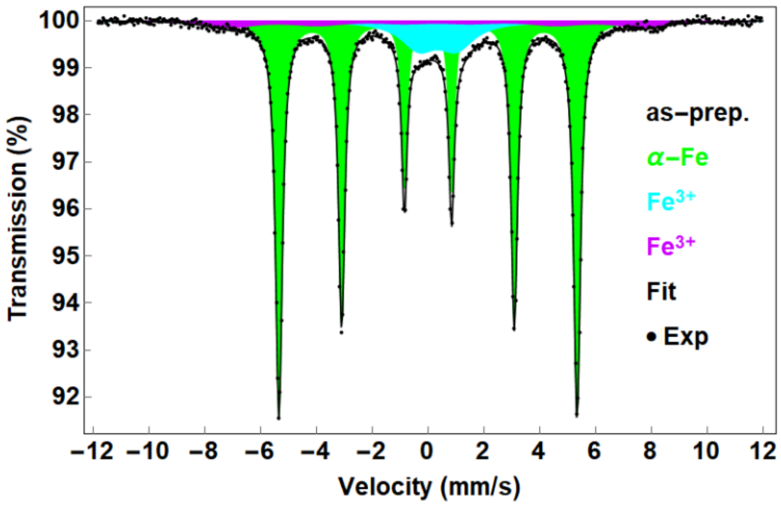

Figure S3. Mössbauer spectrum of the as-prepared zerovalent iron nanoparticles.

Table S2. Hyperfine parameters of the as-prepared zerovalent iron nanoparticles. (IS - isomer shift, QS – quadrupole splitting, FWHM - full width at half maximum, B - hyperfine magnetic field, A – spectrum area).

Sample	Phase	IS (mm/s)	QS (mm/s)	FWMH (mm/s)	B (T)	A (%)
ZVI NPs	α-Fe	0.00 ± 0.01	-	0.28 ± 0.01	33.2 ± 0.5	78 ± 2
	Sextet Fe^{3+}	0.31*	-	2.10 ± 0.20	45.8 ± 0.5	9 ± 2
	Doublet Fe^{3+}	0.34	1.35 ± 0.03	1.53 ± 0.05	-	13 ± 2

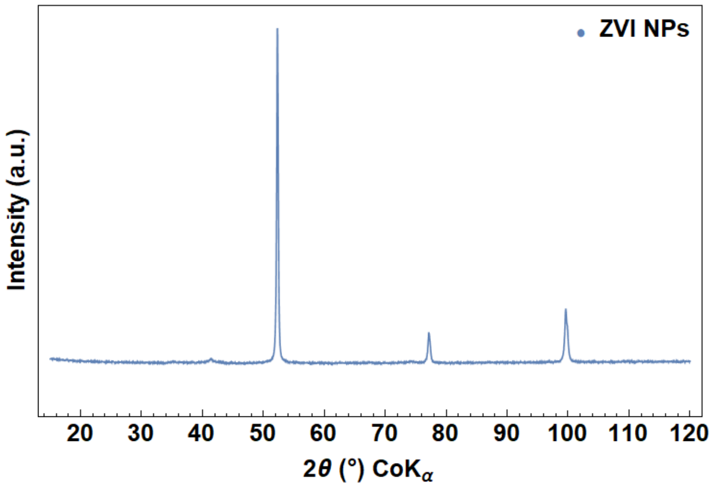

Figure S4. X-ray diffraction pattern of the as-prepared zerovalent iron nanoparticles.

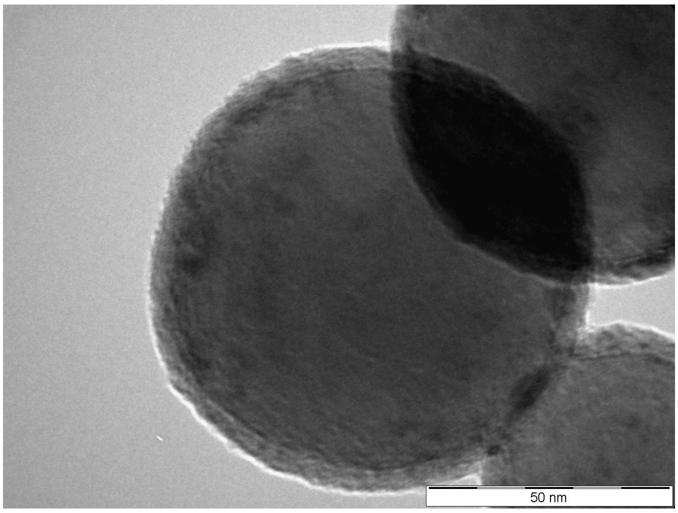

Figure S5. Image of the zerovalent iron nanoparticles from transmission electron microscope.

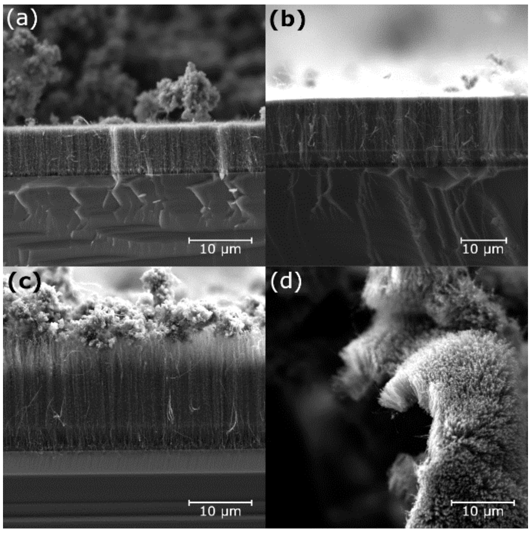

Figure S6. SEM measurement of the products prepared from solid ferrocene. (a) 800 °C – CNTs length: 7.8 µm, (b) 900 °C – CNTs length: 14.3 µm, (c) 1000 °C – CNTs length: 18.0 µm and (d) 1100 °C – CNTs length: 4.7 µm.

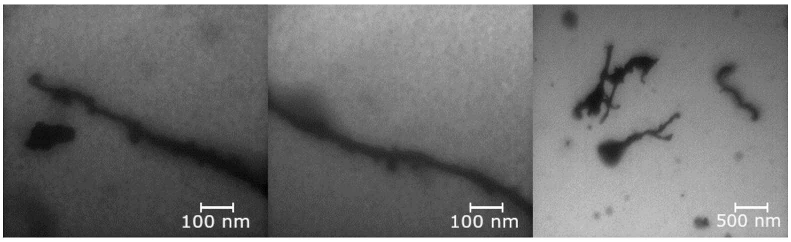

Figure S7. Low resolution TEM measurement of carbon nanotubes prepared by solid ferrocene.
